# Kaspar Schott’s “encyclopedia of all mathematical sciences”

**DOI:** 10.1007/s10202-011-0090-1

**Published:** 2011-05-13

**Authors:** Eberhard Knobloch

**Affiliations:** Technische Universität Berlin, Berlin-Brandenburgische Akademie der Wissenschaften, Berlin, Germany

## Abstract

In 1661, Kaspar Schott published his comprehensive textbook “Cursus mathematicus” in Würzburg for the first time, his “Encyclopedia of all mathematical sciences”. It was so successful that it was published again in 1674 and 1677. In its 28 books, Schott gave an introduction for beginners in 22 mathematical disciplines by means of 533 figures and numerous tables. He wanted to avoid the shortness and the unintelligibility of his predecessors Alsted and Hérigone. He cited or recommended far more than hundred authors, among them Protestants like Michael Stifel and Johannes Kepler, but also Catholics like Nicolaus Copernicus. The paper gives a survey of this work and explains especially interesting aspects: The dedication to the German emperor Leopold I., Athanasius Kircher’s letter of recommendation as well as Schott’s classification of sciences, explanations regarding geometry, astronomy, and algebra.

## Abstract (de)

1661 veröffentlichte Kaspar Schott sein umfassendes Lehrbuch “Cursus mathematicus” in Würzburg zum ersten Mal, seine “Enzyklopädie aller mathematischen Wissenschaften”. Es war so erfolgreich, dass es 1674 und 1677 erneut veröffentlicht wurde. In dessen 28 Büchern gab Schott eine Einführung für Anfänger in 22 mathematische Disziplinen mit Hilfe von 533 Abbildungen und zahlreichen Tafeln. Er wollte die Kürze und Unverständlichkeit seiner Vorgänger Alsted und Hérigone vermeiden. Er zitierte oder empfahl weit mehr als hundert Autoren, unter ihnen Protestanten wie Michael Stifel und Johannes Kepler, aber auch Katholiken wie Nicolaus Copernicus. Der Aufsatz gibt einen Überblick über dieses Werk und erklärt besonders interessante Aspekte: Die Widmung für den deutschen Kaiser Leopold I., Athanasius Kirchers Empfehlungsschreiben ebenso wie Schotts Klassifikation der Wissenschaften und Erklärungen zur Geometrie, Astronomie und Algebra.

## Résumé

En 1661, Kaspar Schott publia son manuel compréhensif “Cursus mathematicus” à Würzburg pour la première fois, son “Encyclopédie de toutes les sciences mathématiques”. Il était tellement couronné de succès qu’il fut publié de nouveau en 1674 et en 1677. Dans ses 28 livres, Schott donna une introduction au moyen de 533 figures et de nombreuses tables. Il voulait éviter la brièveté et l’incompréhensibilité de ses prédecesseurs Alsted et Hérigone. Il citait ou recommendait de loin plus de cent auteurs, entre autres des protestants comme Michael Stifel et Johannes Kepler, mais aussi des catholiques comme Nicolas Copernic. L’article donne une vue d’ensemble de cette oeuvre et explique des aspects particulièrement intéressants: La dédicace à l’empereur allemand Leopold I, la lettre de recommendation d’Athanase Kircher de même que la classification des sciences de Schott et ses explications géométriques, astronomiques et algébriques.

## Introduction

Since Plato’s time everybody agrees that geometry is of utmost importance, the American Mathematical Society included. It was founded in 1888. Its seal bears the date ‘1923’ and shows a Greek temple together with the famous Greek inscription attributed to Plato’s academy: " " (“Nobody should enter who does not know geometry!”) (Illustration 1).

**Illustration 1:**
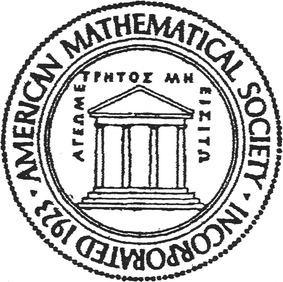
Seal of the American Mathematical Society

The temple represents a religious context. Geometry is indispensable in the *kallipolis* (beautiful city) of Plato’s *State*. The German Association of Mathematicians (Deutsche Mathematiker-Vereinigung) was founded in 1890. Since 1902, it has had its own seal: (Illustration 2).

**Illustration 2:**
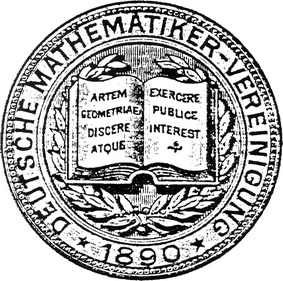
Seal of the Deutsche Mathematiker-Vereinigung

An opened book shows the Latin inscription: “Artem geometriae discere atque exercere publice interest” (“It is in public interest to learn and to practise the art of geometry”). (Knobloch 2001:34). Yet, where does this inscription come from? It has been taken from the Roman corpus of laws (Codex Iustinianus Book 9, Chap. 18:§2). The chapter is entitled: “De maleficis et mathematicis et ceteris similibus” (“On wrongdoers and mathematicians and the others similar to them”).

In the first paragraph, it is stated that it is worse to extinguish a man by poison than to kill him with a sword. This seems to be a really astonishing context for geometry. The second paragraph reads: “Artem geometriae discere atque exerceri publice intersit. ars autem mathematica damnabilis interdicta est.” The first sentence is—in principle—the inscription of the German seal. The second sentence just means: “But the damnable mathematical art is forbidden.” At last the reader now recognizes that the mathematicians in this juridical text of the sixth century A.C. are the astrologers. The mathematical art is astrology.

When in 1661 the German Jesuit Kaspar Schott published his mathematical textbook “Cursus mathematicus” he addressed both aspects of mathematics or—more specifically—of geometry: its great utility and the religious ideas. For him, arithmetic and geometry were the wings for the beginner (tyro) in order to penetrate the sanctuaries of mathematics (mathematicae adyta). A sanctuary (adytum) is the innermost part of a temple, strictly speaking what must not be entered, as in the case of the American Mathematical Society seal. In other words, from the very beginning, Schott put his work into a religious context.

I would like to give a survey of this impressing work by dealing with four issues: (1) The work, its dedication and eulogies, (2) conception, (3) realization, (4) examples taken from the chapters on geometry, astronomy, and algebra.

## The work, its dedication and eulogies

First of all, the title page is worth studying more diligently: (Illustration 3).

**Illustration 3:**
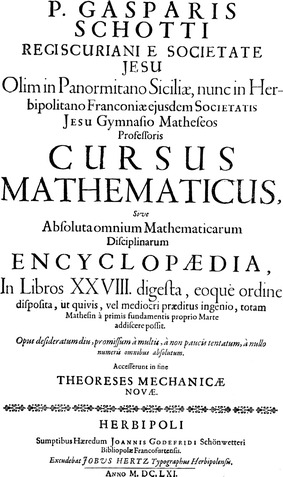
Title page of Kaspar Schott’s *Cursus mathematicus*

The Latin title reads in English: “Mathematical course of perfect encyclopedia of all mathematical disciplines of father Kaspar Schott, born in Königshofen belonging to the Society of Jesus, professor of mathematics once at the University of Palermo in Sicily, now at the Franconian University of Würzburg of the same Society of Jesus. It is divided into 28 books and arranged in such an order that everybody even if he should be equipped with mediocre talents can independently learn the whole of mathematics, beginning with the first foundations. A work desired for a long time, promised by many, tried by not few, perfectly carried out by nobody. At the end new mechanical theorems are added.”

Without question, Schott’s claim is self-conscious and confident. We shall see later on whether Schott came up to his own expectations.

The copperplate print, also reproduced in Remmert (2005:213), is of special interest: (Illustration 4).

**Illustration 4:**
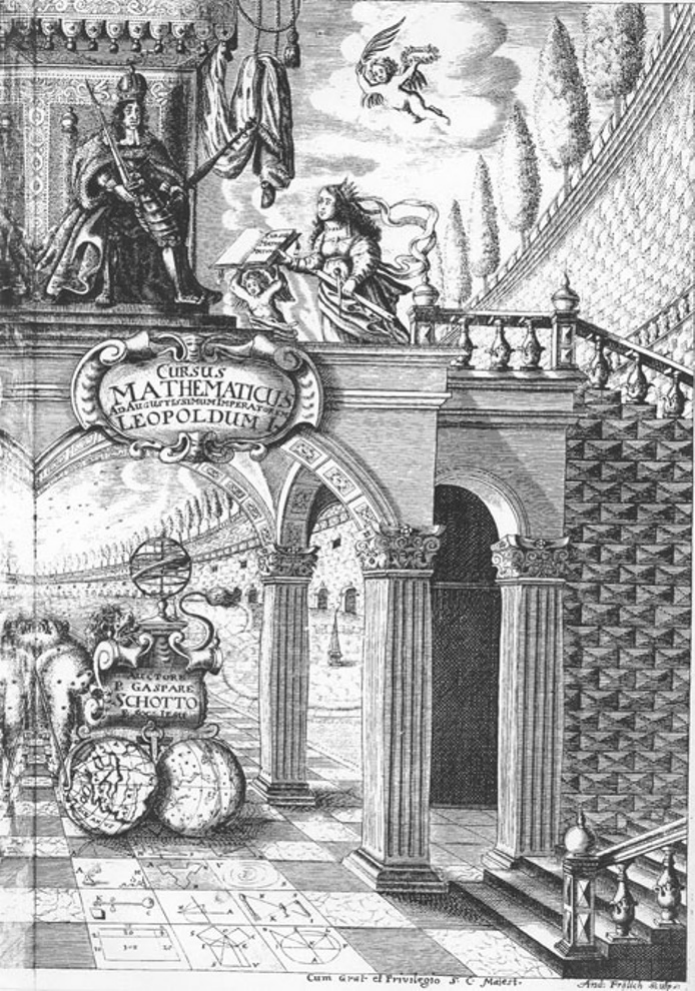
Copperplate print of Kaspar Schott’s *Cursus mathematicus*

It shows the hardly 21-year-old emperor Leopold I. with sword and scepter. In 1658, 3 years earlier, he had been crowned German king and Holy Roman emperor in Frankfurt/Main. Schott alludes to this event in the following dedication to Leopold I. His plan to write the “Cursus mathematicus” (Mathematical course) dates back to that coronation year. A crowned lady with a pair of compasses and a ruler in her left hand offers the “Cursus mathematicus” to the emperor. This emperor highly esteemed the arts and sciences and was himself a gifted musician in spite of the many wars he had still to carry on. Thanks to the insignia, the lady can be undoubtedly identified with geometry. Its crown underlines its importance. An angel holds a laurel wreath that was meant for the author of the work. We will come back to this issue in the epilogue.

On the ground-floor, a strange vehicle is drawn by the true constellations Bear and Lion. They draw the terrestrial and the celestial globes that is the vehicle has two ‘wheels’ as in the case of Roman chariots. The armillary sphere that is a world model replaces the driver. The globes or spheres are symbolizing Schott’s *mathesis* that is moved along below the emperor as is stated in the dedication (*advolvitur*). The course is originally a running, a race track.

The globes reveal 12 tilings of the ground-floor that is paved by squares. Nine of them can be easily identified (from the left to the right, from above to below): ballistics (warfare), military architecture (fortification), Tychonic world system, statics, gnomonics, practical geometry, algebra, geometry, and trigonometry. Obviously, the interests of an emperor being at war, shown in the armor of a knight, have influenced the selection of these nine represented mathematical disciplines out of the 22 dealt with in the volume.

Utility is the leading aspect. It is not by chance that Schott will call practical geometry the noblest and most useful among all mathematical disciplines, trigonometry necessary and useful in the highest degree, gnomonics even divine.

The dedication to Leopold I. contains some important hints at the work: “Regi encyclopaediam mathematicam o(ffert) d(at) c(onsecrat) Casparus Schott” (“Kaspar Schott offers, endows, consecrates the mathematical encyclopedia to the king.”).

Encouraged by the emperor, Schott says, he has covered the immeasurable sea of mathematical disciplines (“immensum mathematicarum disciplinarum pelagus…percurrerem”) (Schott 1661:p.)(2v°) within one year that is he has carried out the course. This is indeed an astonishing achievement because the volume comprehends 700 folio pages and 533 figures. This time Schott uses the popular metaphor employed for example by Francis Bacon before him and by Gottfried Wilhelm Leibniz after him: To pursue, science is something like a voyage of discovery.

According to Schott, the emperor knows that the mathematical sciences, combined with the experience of philosophy, are jewels in the hands of princes, kings, and emperors. Kings and monarchs of the world always attached great interest to the mathematical sciences. One might think of Alphonse the Wise of Castile or Charles the Great. Without question, in the eyes of the princes of the seventeenth century mathematics was a discipline supporting the state, it was powerful.

This is demonstrated for example by Daniel Bretschneider the Elder, citizen and painter in Dresden by his “Book about various inventions” (“Ein Buch von allerley Inventionen”): (Illustration 5).

**Illustration 5:**
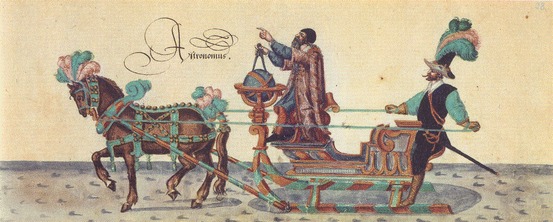
A sleigh with an astronomer taken from Daniel Bretschneider’s *Ein Buch vonallerley inventionen* (1602)

An astronomer is standing with commanding air on a sleigh bearing the garment of a sovereign and measures the celestial globe. He looks like God. And indeed, in the Middle Ages, God was painted in a similar pose as the highest geometer.

Schott is especially proud of his dedication. It is true that most of the eminent mathematicians of all times have dedicated their works to princes and sovereigns. Johannes Kepler dedicated his “Harmonice mundi” (“World harmony”) to the English king James, his “Astronomia nova” (“New astronomy”) and his “Astronomiae pars optica” (“Optical part of astronomy”) to the emperor Rudolph II., his “Tabulae Rudolphinae” (“Rudolfinian tables”) to the emperor Ferdinand II., the father of Leopold I.: A similar behavior is true of Clavius, Kircher, and Grégoire de Saint Vincent. But up to then nobody has dedicated a whole encyclopedia of the mathematical sciences to a sovereign. Schott’s citation of Kepler’s works should be all the more emphasized because they do not play any role in the “Mathematical course”.

Schott had inherited his yearning for universality from his Lullistic teacher Athanasius Kircher. Already his “Magia universalis naturae et artis” (“Universal magic of nature and art”) whose four volumes were published between 1657 and 1659 in Würzburg mirrors this claim. And indeed, the seventeenth century is the century of universal mathematics. Kircher called his works “Musurgia universalis” (“Universal art of music” 1650), “Polygraphia nova et universalis” (“New and universal art of writing much” 1663). On the title page of his “Ars magna sive combinatoria” (“Great or combinatorial art” 1670) the maxim was to be read: " " (“Nothing is more beautiful than to know everything.”).

While Schott was elaborating his encyclopedia, he was encouraged by many contemporaries. He received *paraeneses*, exhortations. He printed three of them at the beginning of his volume. One of these writings was the letter of his admired teacher Athanasius Kircher to mathematical beginners. Kircher wrote a classical *enkomium*, a eulogy on mathematics as we meet it again and again in the Renaissance, especially in Johannes Regiomontanus’s famous inaugural address in Padua from 1464.

Kircher explains that in his dialog “Symposion” Plato had Alcibiades speak of Socrates as a pharmacist’s pot. He, Kircher, preferred to compare the studies of mathematics with a workshop (*officina*). For a long time, the mathematical community (*res publica mathematica*) has waited for an author like Schott who would write a “Mathematical course”, an encyclopedia of the whole mathematics, clearly structured and based on an easy method in order to foster the scientific community (*res publica litteraria*) and to encourage beginners.

Kircher speaks about the comprehensive use of the divine mathematics (*sacra mathesis*) (Schott 1661, p.)()(4r°), about the divine studies of mathematics (*divinum matheseos studium*) that alone imparts recognition and knowledge of all the celestial things:

“Sunt haec divinae Matheseos miracula; adeo ut nihil inter humanae mentis reperta sit tam rarum, mirum, insolitum, paradoxum, quod Mathesis non ex abditis Arithmeticae, Geometriae, Musicae, Astronomiae, Opticae, Mechanicae, Staticaeque officinis, miro quodam Artis et Naturae conjugio produxerit.” (“These are the miracles of the divine mathematics to such a degree that nothing is so rare, wounderful, unusual, paradoxical among the inventions of human mind that mathematics did not bring about it from the hidden workshops of arithmetic, geometry, music, astronomy, optics, mechanics, and statics by a certain miraculous union of art and nature.”).

There cannot be any doubt: Kircher and Schott following him gained their explanations from the “Prolegomena”, the preface that Christoph Clavius, chief mathematician of the Jesuits, had placed in front of his edition of Euclid’s “Elements”. God himself was the first geometer who had created the world according to arithmetical and geometrical principles.

Knowledge about the world presupposed the language of mathematics. Regularity and perfection of the unchangeable, geometrical structure of the world testified for its divine origin (Romano 1999:133–178). This theological view established for Clavius the importance of mathematics before all other disciplines, even before philosophy and theology.

God was the inventor of all mathematical disciplines. Schott explicitly said it in his “Horographia” (“Gnomonics”) (Schott 1661:389): “Persuasissimum mihi semper fuit, tum omnes Mathematicas Disciplinas, tum vero maxime Gnomonicam, seu Horographiam…Inventorem alium non agnoscere, quam DEUM Optimum Maximum. Haec ergo vere divina scientia…” (“I was always completely convinced that all mathematical disciplines, but especially gnomonics or the art of making sun-dials, acknowledge as inventor none but the greatest, best God. Hence this truly divine science…”).

This importance compensates by far for the trouble of acquiring it because: " ." (“The gods grant everything on the strength of pains.”). Kircher cites Epicharmus, the best known poet of the Doric comedy.

## Conception

Schott planned his textbook most carefully. He placed a preface in front of the whole work and in front of every of the 28 chapters as well. Therein he commented upon his aims and methods and defined the single mathematical, partial disciplines: a model of clarity and order!

He addressed beginners and first candidates of mathematics. That he emphasized again and again. Hence, he inserted short introductions into areas or surveys of areas. He referred to further literature for additional studies. More than hundred authors are mentioned, among them Protestants like Michael Stifel or Johannes Kepler, but also Catholics like Copernicus whose theory was still rejected by the Catholic church in Schott’s time.

Schott did not want to be an innovator, any more than Kepler, nor did he want to treat everything exhaustively. Here, if anywhere the wisdom proved to be true: Only when restricted does somebody prove to be a master. On the contrary, thanks to a wise self-restriction, he intended to explain the essentials in an easily comprehensible form for beginners. In his opinion, his two predecessors Johann Heinrich Alsted and Pierre Hérigone had violated this criterion. They were the only authors who had accomplished a similar work before him.

Alsted’s encyclopedia comprehended the whole system of sciences in seven volumes: (Illustration 6).

**Illustration 6:**
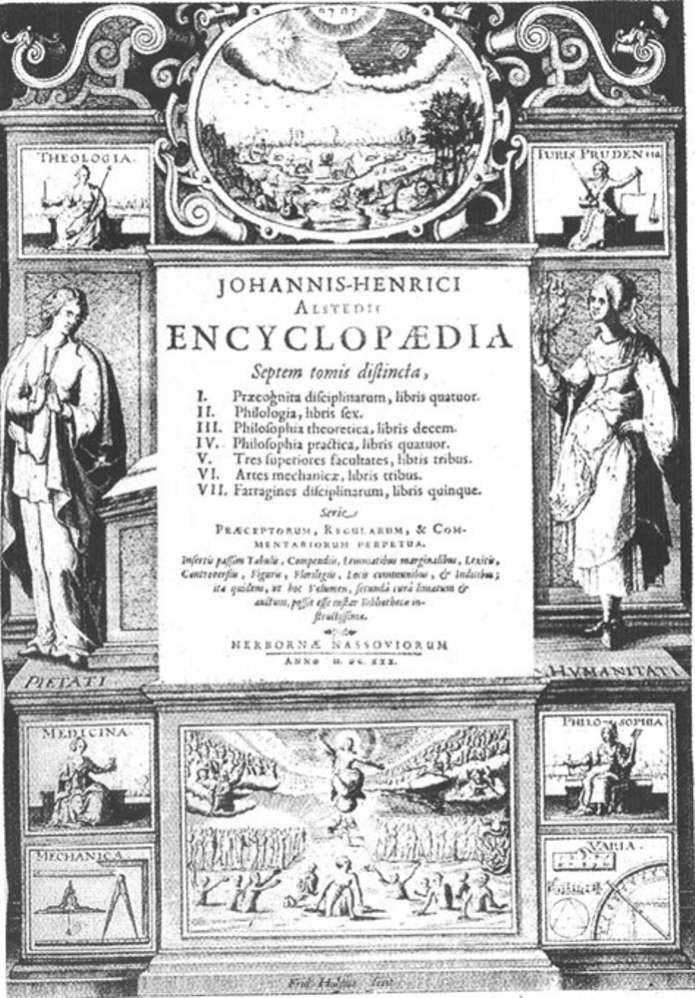
Title page of Johann Heinrich Alsted’s *Enyclopaedia* (1630)

The title page enumerates and illustrates theology, jurisprudence, medicine, philosophy, mechanics, and various subjects (*varia*). Mathematics is subsumed in this last group as it is indicated by the right lower picture and went short for that reason. Arithmetic, music, geometry, and astronomy are represented. Five chapters offered a medley of disciplines (*farragines disciplinarum*).

Pierre Hérigone’s mathematical textbook “Cursus mathematicus” consisted of six volumes that were published in the years 1634–1642. It used artificial symbols as Hérigone proudly emphasized in the title: “Nova, brevi, et clara methodo demonstratus, per notas reales et universales, citra usum cuiuscunque idiomatis, intellectu faciles” (“By a new, short, and clear method demonstrated by means of real and universal signs that can be easily understood without the use of any language.”) (Illustration 7).

**Illustration 7:**
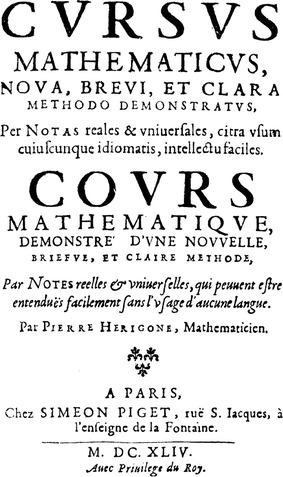
Title page of Pierre Hérigone’s *Cursus mathematicus* (1644)

In the eyes of Schott, however, these symbols make Hérigone’s work too obscure, nearly hieroglyphical. This judgement does not prevent him from often referring to Hérigone.

In particular cases, Schott adheres to clear models, in the case of geometry to Christoph Clavius’ saying (Schott 1661:62): “nemo melius, ordinatius, universalius, clarius, integriusque, ac magis geometrice illo processit” (“nobody proceeded better, in a better order, more universally, more clearly, and more completely, and more geometrically than that one.”).

In the case of astronomy, he adheres to Giovanni Battista Riccioli’s “Almagestum novum” (“New Almagest” 1651) (Schott 1661:261), in the case of music to Athanasius Kircher’s “Musurgia universalis” (Schott 1661:612), in the case of algebra again to Clavius (Schott 1661:527).

He likes to refer to his own books, too, for example to his “Pantometrum Kircherianum” (“Kircher’s instrument for measuring everything” 1660) or to his “Magia universalis naturae et artis”. Its third volume deals with mathematics and its fourth with physics: (Illustration 8).

**Illustration 8:**
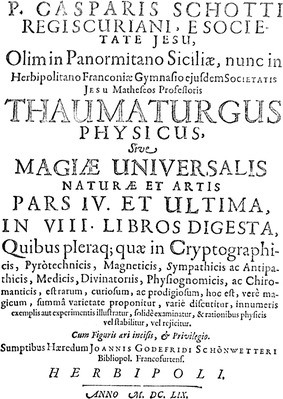
Title page of Kaspar Schott’s *Magia universalis* (fourth part, 1659)

“Thaumaturgus physicus, sive magiae universalis naturae et artis pars IV. et ultima in VIII. libros digesta, quibus pleraque quae in cryptographicis, pyrotechnicis, magneticis, sympathicis ac antipathicis, medicis, divinatoriis, physiognomicis, ac chiromanthicis, est rarum, curiosum, ac prodigiosum, hoc est, vere magicum, summa varietate proponitur, varie discutitur, innumeris exemplis aut experimentis illustratur, solide examinatur, et rationibus physicis vel stabilitur, vel rejicitur.” (“Physical miracle worker or fourth and last part of the universal magic of nature and art, divided into eight books, by which most of what is rare, curious, and prodigious that is truly magic in cryptography, pyrotechnics, magnetism, sympathetic and antipathetic things, medicine, divination, physiognomics, and chiromancy is set forth with greatest variety, is variously discussed, illustrated by innumerable examples and experiments, strongly examined, and confirmed or rejected by physical reasons.”).

Yet, Schott remained a self-critical realist and remarked (Schott 1661, preface of the whole work p.)(5v°): “Quod (sc. consilium) si non assecutus omnino videbor, considera LECTOR, non me hibisco gracili, vel scirpo fiscellam contexere, sed numeris, schematibus, et apodixibus Mathematicis, quae nec Natura, nec Ars protulit sine nodis ac spinis.” (“Should I not seem to have completely reached this aim, note reader that I do not weave a small basket using thin marsh-mallow or bulrush but numbers, figures, and mathematical demonstrations that neither nature nor art brought about without knots and thorns.”).

We will come back to this point. Though he venerated his models, Schott deviated from their methods and opinions in special cases. For example, he rejected Clavius’s demonstration of the parallel postulate (Schott 1661:73) and he rejected Kircher’s belief in trepidation, that is, in a third motion of the earth that does not really exist (Schott 1661:243). In case of doubt he preferred to say nothing instead of nonsense (Schott 1661:522): “Malo itaque nihil dicere, quam tam incerta in medium proferre.” (“For that reason I prefer to say nothing instead of bringing forth such uncertain things.”).

## Realization

At the beginning of the first book that is in his “Isagoge mathematica” (“Mathematical introduction”) Schott gives the traditional definition of mathematics from ancient times (Schott 1661:2): “Ex his colligitur, quae sit Mathematicae essentia seu natura; est quippe Scientia tractans de quantitate terminata, eaque vel a sensibili materia abstracta, vel eidem immersa.” (“From this it is deduced what the essence or nature of mathematics is. It is indeed the science dealing with bounded quantity and this is either abstracted from sensible matter or immersed in it.”).

This definition is still valid in the eighteenth century and occurs in Christian Wolff’s “Mathematisches Lexicon” (“Mathematical lexicon”) dating from 1716 (Wolff 1716:col. 863). Indivisibles however are non-quantities by definition. Already for this reason it is not necessary for Schott to discuss Bonaventura Cavalieri’s indivisibles. Yet, he knows and uses Cavalieri’s logarithmic tables (Schott 1661:605).

In view of numerous classifications of the mathematical disciplines, Schott decides on a classification that is similar to that of Clavius (Schott 1661:3): 

The dichotomic classification principle discrete/continuous, abstract/concrete, speculative/practical is only neglected in the case of concrete, continuous mathematics: music occurs twice. Every subdivision is divided into further subunits. It might suffice to enumerate all 22 disciplines dealt with by Schott: Arithmetic, geometry, trigonometry, astronomy, astrology, chronology, geography, navigation, gnomonics, mechanics, statics, hydrostatics, hydraulic engineering, optics, catoptrics, dioptrics, fortification, warfare, tactics, musical theory, algebra, and theory of logarithms.

Apart from some few differences, this is Hérigone’s list (Folkerts et al. 2001:10). The three chapters bearing upon military subjects are written according to Hérigone’s model. Yet, obviously they also reflect the emperor’s interests. Neither Hérigone nor Schott deal with civil architecture though it occurs in the extended final classification. Alsted had excluded both types of architecture from the mathematical sciences.

The five longest chapters by far (60–92 pages) are geometry, geography, trigonometry, algebra, and astronomy. The shortest chapters (four to six pages) are dioptrics, statics, and tactics. Schott does not deal with theoretical arithmetic that is number theory in the sense of the Euclidean books 7–9, only with practical arithmetic. Hence this chapter comprehends only forty pages. But this first chapter was already published 1 year later, in 1662, as a book on its own and was reprinted many times in a more or less abridged version (Vollrath 2009:XII): (Illustration 9).

**Illustration 9:**
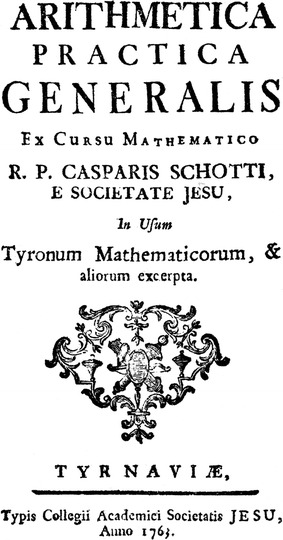
Title page of Kaspar Schott’s *Arithmetica practica* (1763)

The order of the chapters is conditioned by didactical reasons yet also by the dignity of the discipline. Since the biblical history of creation the heavens preceded the earth. The same is true of the corresponding sciences astronomy and geography. Still 200 years later Alexander von Humboldt used this order in his “Kosmos”.

Man imitates the creator. He especially follows God’s example when he describes, constructs the heavens on the earth, when he pursues the “Horographia photo-sciatherica universalis” (“Universal gnomonics or the art of constructing sundials using light and shadow”). Thus, gnomonics is a divine science (Schott 1661:389). This is an old motif. Claudian (ca. 400 A.C.), the last important Latin poet, wrote about the forwardness of Archimedes. The Greek mathematician had constructed a planetary thus imitating Zeus.

At the end of his work, Schott puts the synopsis of all mathematical sciences due to an author whom he is not permitted to mention (Schott 1661:611–620). This time the classification is based on the dichotomy *pura/mixta* (pure/mixed), not on *discreta/continua*. The following schemes represent parts of this comprehensive classification: 

Mixed mathematics is subdivided in the following way: 

The disciplines are again subdivided. Astronomy might serve as an example: 

## Examples taken from the chapters on geometry, astronomy, and algebra

### First example: geometry

Many editions of Euclid’s *Elements* like those of Johann Vögelin, Joachim Camerarius, Jan Pietersz, Henric Coets restricted themselves to the first six books (Folkerts, Knobloch, Reich 2001, 53–65). Schott did the same in his “Geometria elementaris sive Elementorum geometricorum Euclidis sex libri primi” (“Elementary geometry or the six first books of Euclid’s geometrical Elements”).

He believed that he had solved the famous parallel problem in the sense that he had shown that the parallel postulate is a demonstrable theorem (Schott 1661:73) “tametsi id aliter et multis demonstrare conetur Proclus, Clavius, Tacquet et alii.” (“though Proclus, Clavius, Tacquet and others tried to demonstrate this in another way and with a great deal of energy.”). How did he succeed in doing this? The parallel postulate reads as follows: (Illustration 10).

**Illustration 10:**
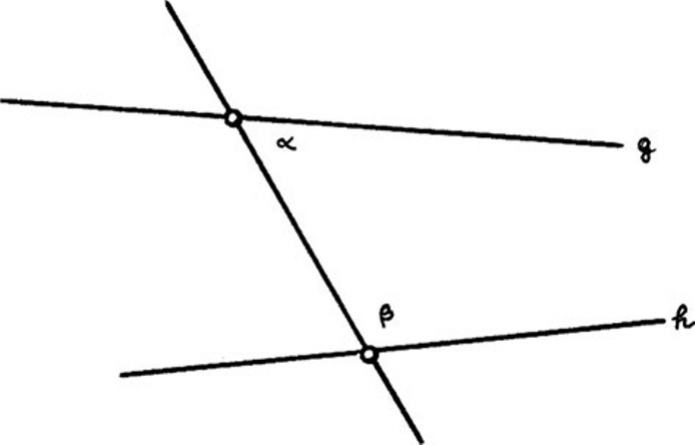
Two straight lines that are not parallel cut by a third straight line

“If the sum of the two angles α and β is smaller than two right angles, the straight lines g, h have a point of intersection on the side of these two angles.” The inversion reads: “If the straight lines g, h have a point of intersection, the sum of the two angles α and β is smaller than two right angles.” The inversion can be indeed demonstrated and is equivalent with theorem I, 28 of Euclid’s “Elements”: (Illustration 11).

**Illustration 11:**
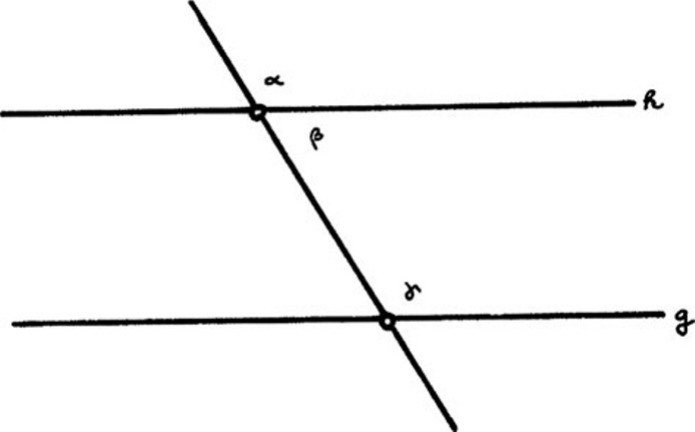
Two straight lines that are parallel cut by a third straight line

“If the sum of α and β is equal to two right angles, the straight lines g, h are parallel.” Hence Schott believes: If the cutting straight line yields two angles so that their sum is smaller than two right angles, the two straight lines must meet on this side. In other words, he infers from

This is a gross logical mistake. Clavius’s mistake is more subtle, but it also necessarily implies a pseudo-demonstration of the postulate.

Practical geometry means measuring, calculation of lengths, surfaces, volumes of lines, areas, solids, wine casks of spherical surfaces, division of areas or geodesy, and metamorphoses. In his practical geometry, Schott explicitly restricts himself to two instruments, to the geometrical square and to the quadrant: (Illustration 12).

**Illustration 12:**
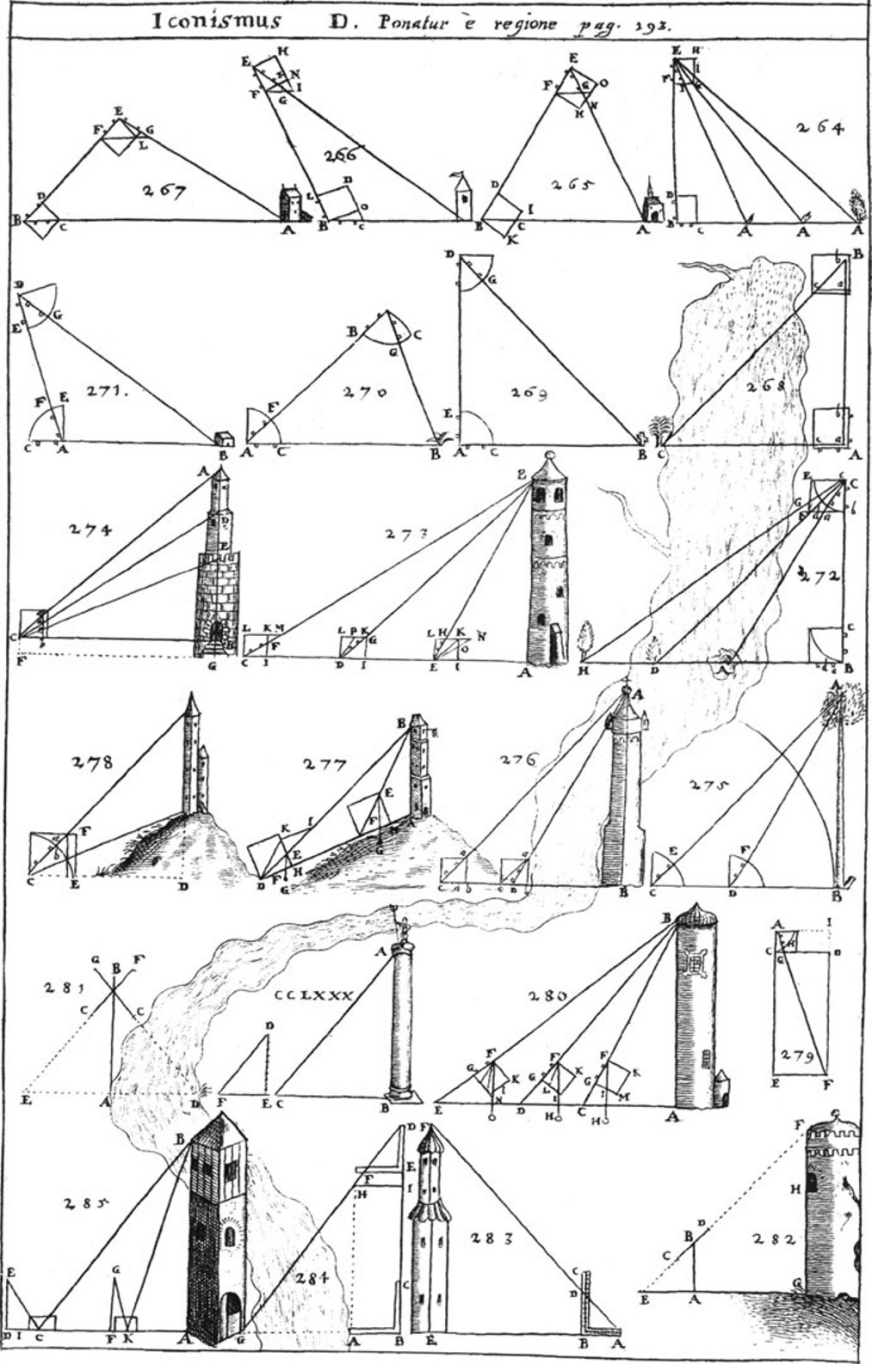
Twenty-two measurings of distances and heights taken from Kaspar Schott’s *Cursus mathematicus*

Many figures illustrate how indirect measuring of distances can be carried out by means of these instruments, for example the height of a tower, the width of a river, the depth of a well. The quantity looked for is calculated by means of one or two measurements or positions and similar triangles (fig. 280 of Illustration 12): The height AB is a multiple of the distance CD between the two positions C, D of measuring.

### Second example: astronomy

In theoretical astronomy, Schott explains six world systems: (Illustration 13).

**Illustration 13:**
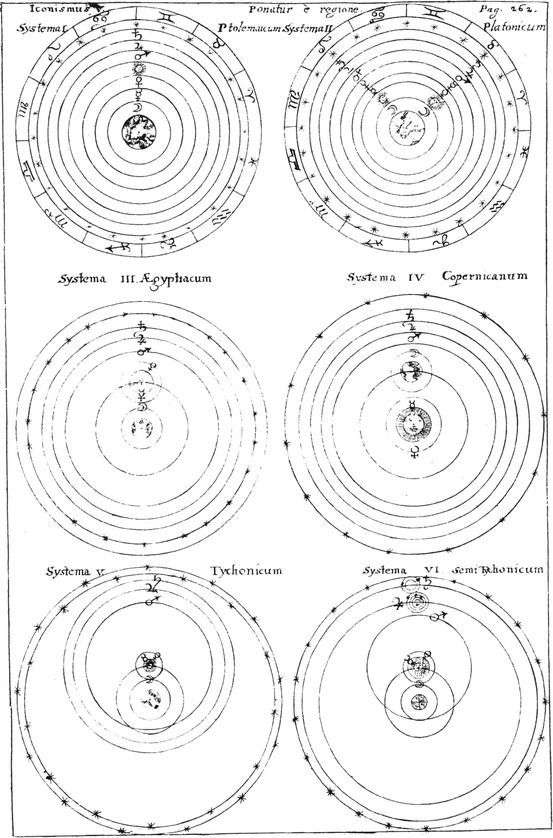
Six world systems taken from Kaspar Schott’s *Cursus mathematicus*

- The geocentric Ptolemaic system: The order of the ‘planets’ is moon, Mercury, Venus, sun, Mars, Jupiter, and Saturn. The sphere of the fixed stars marks the boundary of the finite world.
- The geocentric Platonic system: The order of the ‘planets’ is moon, sun, Mercury, Venus, Mars, Jupiter, and Saturn. The sphere of the fixed stars marks the boundary of the finite world.
- The Egyptian system: The sun turns around the earth, Mercury and Venus about the sun. All the other ‘planets’ moon, Mars, Jupiter, and Saturn turn around the earth. The sphere of the fixed stars marks the boundary of the finite world.
- The heliocentric Copernican system: The planets have no own motion, their spheres turn around the sun. The sphere of the fixed stars is farther away from the centre than in the other world systems.
- The Tychonic system: The heavens of the fixed stars have a certain depth. Moon and sun turn around the earth, all other planets around the sun. The spheres have been abolished. Jupiter has four companions (comites), Saturn has two.
- The semi-Tychonic system: There is no depth of the heavens. Moon, sun, Jupiter, and Saturn turn around the earth. Mercury, Venus, and Mars turn around the sun.

In his *Astronomia elementaris sive de sphaera mundi* (*Elementary astronomy or on the sphere* o*f the world*), Schott defends geocentrism concluding (Schott 1661:242):

Haec sunt praecipua argumenta, quae tametsi evidenter rem propositam non probant, adeo tamen probabilem reddunt, ut temerarius videri queat qui sine evidenti ratione eam negat.

These are the main arguments that—even if they do not evidently prove the proposed affirmation—make it yet probable to such a degree that anyone who refuses it without any evident reason would appear inconsiderate.

Schott was right in saying this because only in 1727 did James Bradley discover the aberration that could be taken as a proof of heliocentrism. Schott himself adheres to Tycho’s system. There are fluid heavens belonging to certain regions. At the University of Mainz, Tycho’s system was taught by the Jesuit Otto Cattenius during the winter term 1610/1611 (Krayer 1991). Schott does not mention Kepler’s laws. He rightly numbers Kepler among the heliocentrists saying (Schott 1661:243): “…quotquot aliis prurit animus et calamus ad nova.” (“…among all those whose mind and pencil have the itch to do new things.”). Actually Kepler has defended himself against this reproach using the same expression *prurigo* (itch) (Kepler 1618–1621:254f.; Knobloch 1997).

In principle, Schott explains the Ptolemaic planetary theory (Figs. 340–346 of Illustration 14) that was based on excentric deferents, epicycles, equants (puncta aequantia) saying (Schott 1661:273): (Illustration 14).

**Illustration 14:**
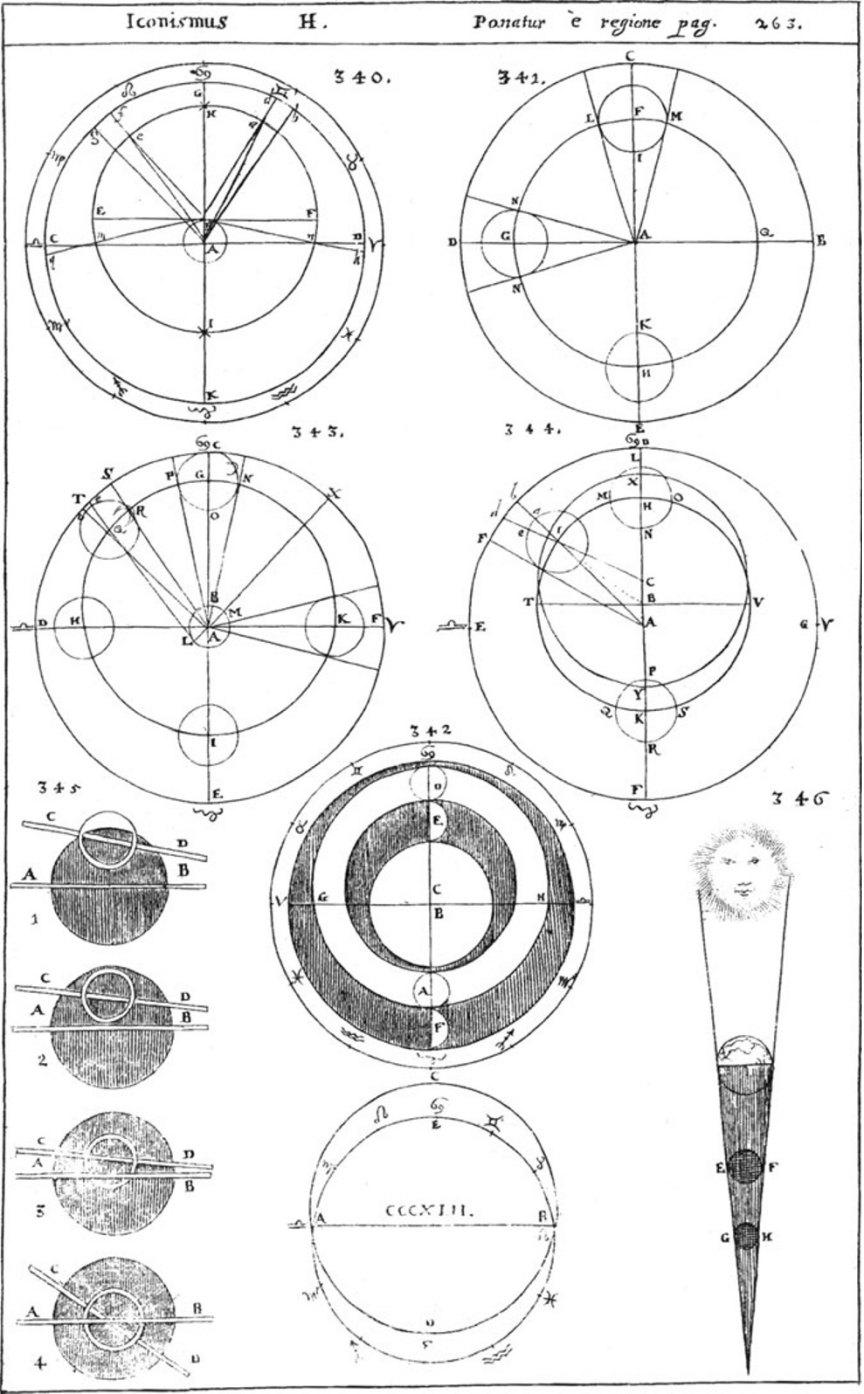
Ptolemy’s planetary theory, explanation of eclipses taken from Kaspar Schott’s *Cursus mathematicus*

“Hac hypothesi putabat Ptolemaeus sufficienter salvari supradicta phaenomena.” (“Ptolemy believed that the phaenomena mentioned above are sufficiently saved by this hypothesis.”).

### Third example: algebra

The penultimate chapter deals with algebra. Some people dare to call it divine, Schott says, because of its subtlety (*subtilitas*) and excellence (*excellentia*) (Schott 1661:526). This is no longer astonishing for us because all mathematical disciplines have a divine origin as we have heard. Schott uses the symbolism that was derived from the Cossic tradition and applied by Clavius, for example Rq for ‘square root’. He explains the calculation with algebraic quantities. With regard to equation theory, he refers to François Viète, Pierre Hérigone, and Michael Stifel. Still Gottfried Wilhelm Leibniz tried to free people from their fear of algebra. To this end, he elaborated a dialog between a teacher and a boy (Leibniz 1976).

### Epilogue

There were a second and a third edition of Schott’s textbook that appeared in 1674 and in 1677, respectively: The volume was a great success. In the seventeenth and eighteenth centuries, many similar mathematical encyclopedias followed, also by Jesuits like that of François Milliet Dechales. The mathematical method achieved great triumphs, especially in Christian Wolff’s “Elementa matheseos universae” (“Elements of the whole mathematics”) (Folkerts, Knobloch, Reich 2001:18–29).

Schott himself remained modest saying (Schott 1661:660): “Nullam tamen a quopiam, praeterquam a munificentissimo remuneratore DEO, victoriae lauream exspecto, quoniam numeris omnibus absolutum Opus, quale Lector exspectabat, non exhibeo…Hic interim, ad DEI OPTIMI MAXIMI gloriam, Reique publicae Litterariae utilitatem, quam utramque prae oculis unice habui semper, esto totius cursus mathematici finis.” (“Yet, I do not expect victor’s laurel from anybody other than God, the most munificent recompenser, because I do not present a work perfect throughout as the reader expected it…Meanwhile here the end of the whole mathematical textbook should be to the glory of the highest, best God and to the use of the scientific community that I always thought of exclusively.”).

The Polish Jesuit and Professor of Mathematics Adam Adamandus Kochanski had sent 47 Alcaic Strophes to Schott. This meter had been introduced into Roman poetry by Horace. Such a strophe comprehends two verses, each of eleven syllables, a third consisting of nine, and a fourth verse consisting of ten syllables. Hence Kochanski spoke of Horace’s lyre. The last strophe reads (Schott 1661, p.)()(6v°):

Hanc linque Sphyngem barbytos Oedipo;

Vatumque sacro percitus entheo

Futura praedic. HIC PERENNES

CURSUS AGET TIBI SCHOTTE LAUDES.

Leave this sphinx, lyre, to Oedipus;

Foretell the future excited by the

inspired holy action of the prophets. This

textbook will bring you eternal glory.

Nothing has to be added to this statement.

## Appendix: Illustration credits

Illustration 1: Raymond Clare Archibald, *A semicentennial History of the American Mathematical Society 1888*–*1938 with Biographies and Bibliographies of the Past Presidents*. New York 1938, reverse of the title page.

Illustration 2: *Jahresbericht der Deutschen Mathematiker*-*Vereinigung* 11 (1902), title page.

Illustration 3: Kaspar Schott, *Cursus mathematicus*. Würzburg 1661, title page (Herzog August Bibliothek Wolfenbüttel: diglib.hab.de).

Illustration 4: Kaspar Schott, *Cursus mathematicus*. Würzburg 1661, copperplate print (diglib.hab.de).

Illustration 5: Michael Korey, *Die Geometrie der Macht, Die Macht der Geometrie,**Mathematische Instrumente und fürstliche Mechanik um 1600 aus dem Mathematisch*-*Physikalischen Salon*. München-Berlin 2007, 5.

Illustration 6: Menso Folkerts, Eberhard Knobloch, Karin Reich (eds.), *Mass, Zahl und Gewicht, Mathematik als Schlüssel zu Weltverständnis und Weltbeherrschung.* 2nd ed. Wolfenbüttel 2001, 14.

Illustration 7: Menso Folkerts, Eberhard Knobloch, Karin Reich (eds.), *Mass, Zahl und Gewicht, Mathematik als Schlüssel zu Weltverständnis und Weltbeherrschung*. 2nd ed. Wolfenbüttel 2001, 14.

Illustration 8: Kaspar Schott, *Magia universalis naturae et artis*, Part IV. Frankfurt 1659 (Staatsbibliothek zu Berlin—Preußischer Kulturbesitz).

Illustration 9: Hans-Joachim Vollrath (ed.), *Kaspar Schotts Rechenbüchlein*, Faksimile und Übersetzung. Würzburg 2009, 3.

Illustration 10: Benno Artmann, *Euclid*—*The Creation of Mathematics*. New York, etc. 1999, 33.

Illlustration 11: Benno Artmann, *Euclid*—*The Creation of Mathematics*. New York, etc. 1999, 33.

Illustration 12: Kaspar Schott, *Cursus mathematicus*. Würzburg 1661, after page 192 (see Illustration 3).

Illustration 13: Kaspar Schott, *Cursus mathematicus*. Würzburg 1661, after page 262 (see Illustration 3).

Illustration 14: Kaspar Schott, *Cursus mathematicus*. Würzburg 1661, before page 263 (see Illustration 3).
